# Supplementary material for: Nanocomposites Based on Antiferroelectric Liquid Crystal (S)-MHPOBC Doping with Au Nanoparticles
Source: Molecules. 2022 Jun 7;27(12):3663. doi: 10.3390/molecules27123663 (PMC9230379; doi:10.3390/molecules27123663)
Supplement: Supplementary file 1 [file molecules-27-03663-s001.zip › molecules-1727615-supplementary.pdf]

Supplementary Materials

Composite 1	Composite 2	Composite 3

SmA* (125°C)

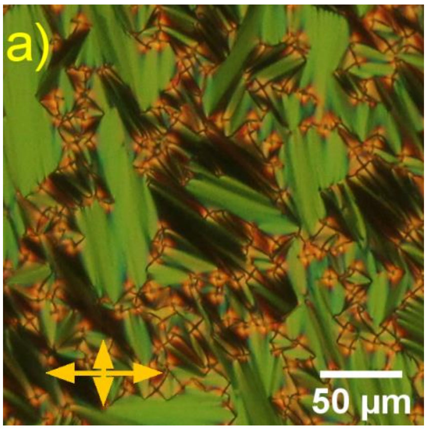

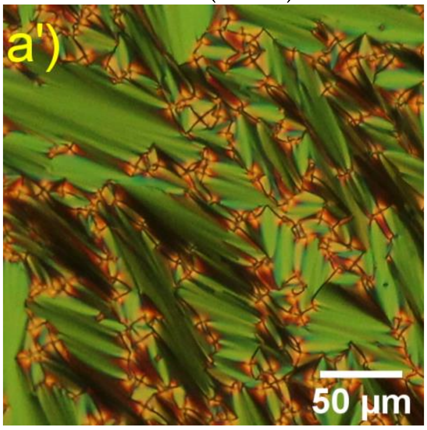

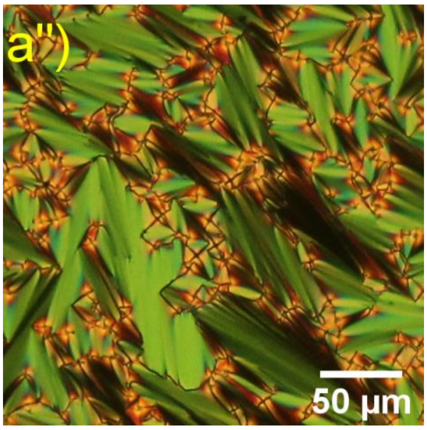

SmC*$_{\alpha}$ (117°C)

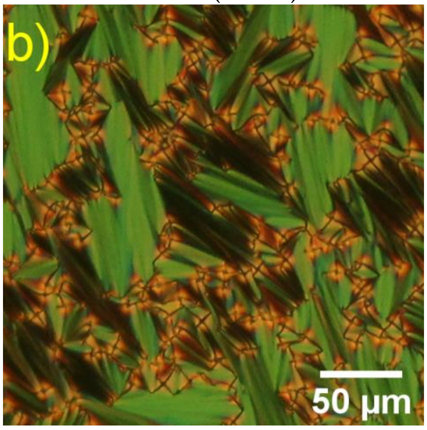

SmC*$_{\alpha}$ (118°C)

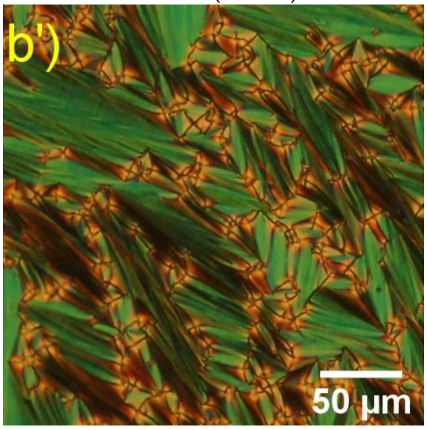

SmC*$_{\alpha}$ (118°C)

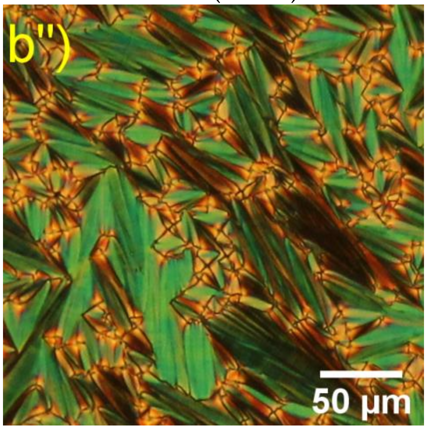

SmC* (116°C)

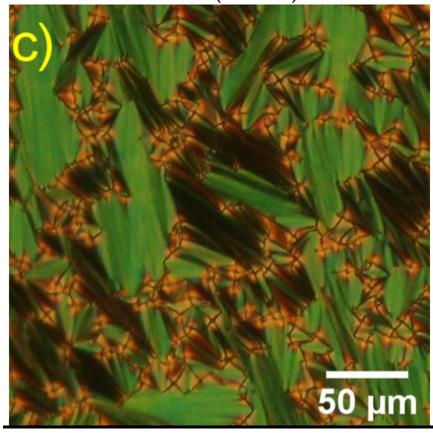

SmC* (117°C)

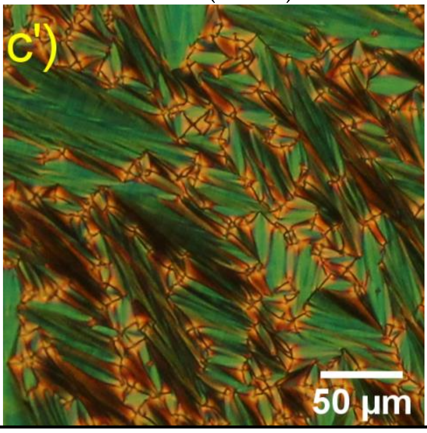

SmC* (117°C)

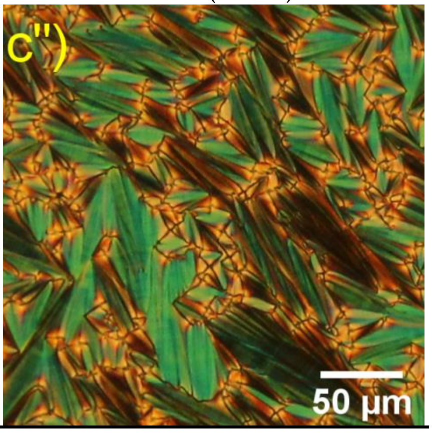

SmC*$_{\gamma}$ (114°C)

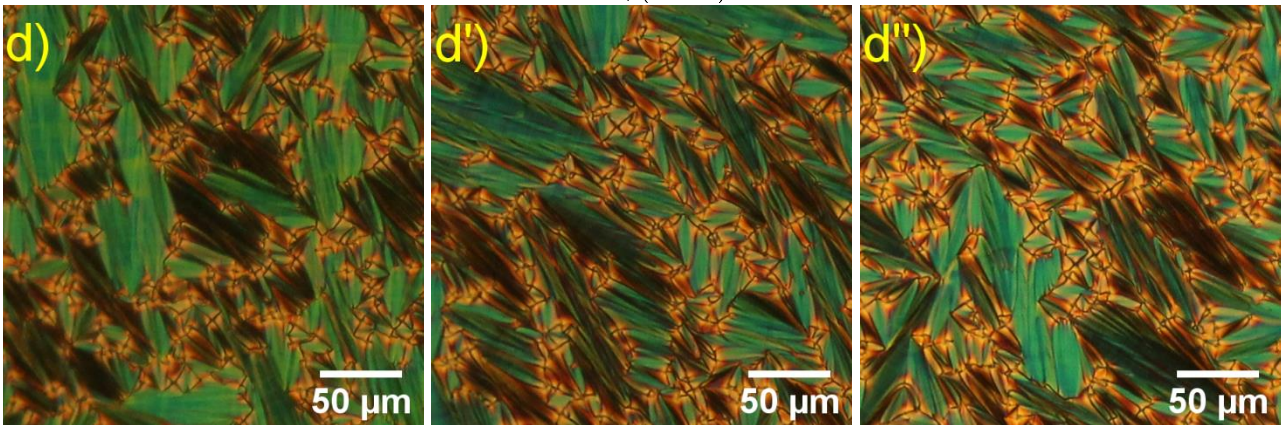

SmC*$_{A}$ (105°C)

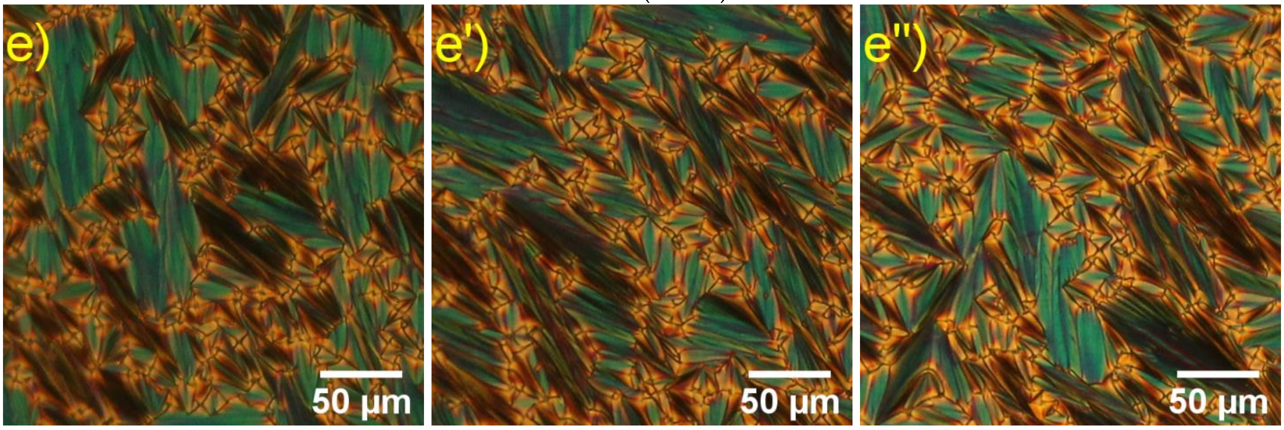

SmC*$_{A}$ (90°C)

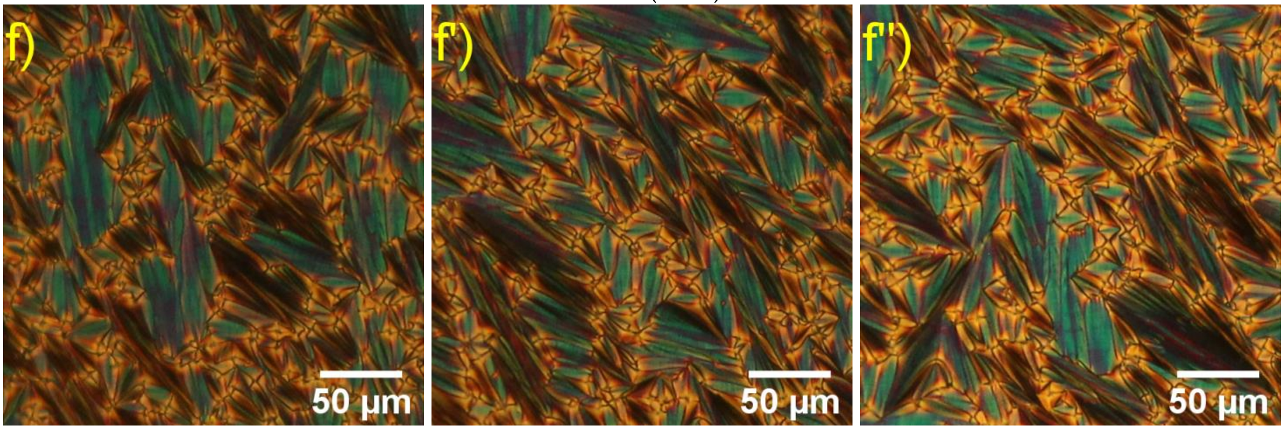

SmI* (60°C)

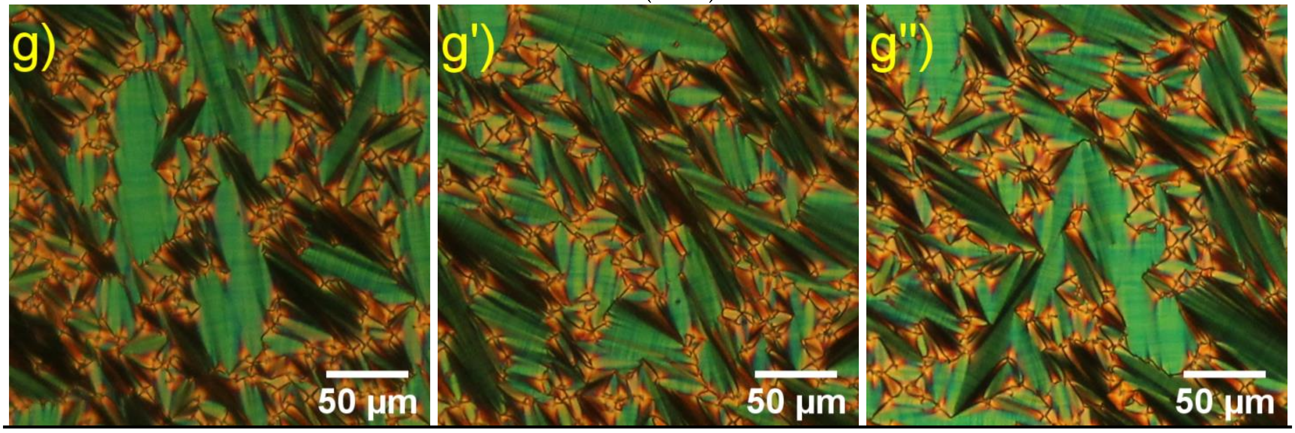

Coexistence SmI*+Cr1 (40°C)

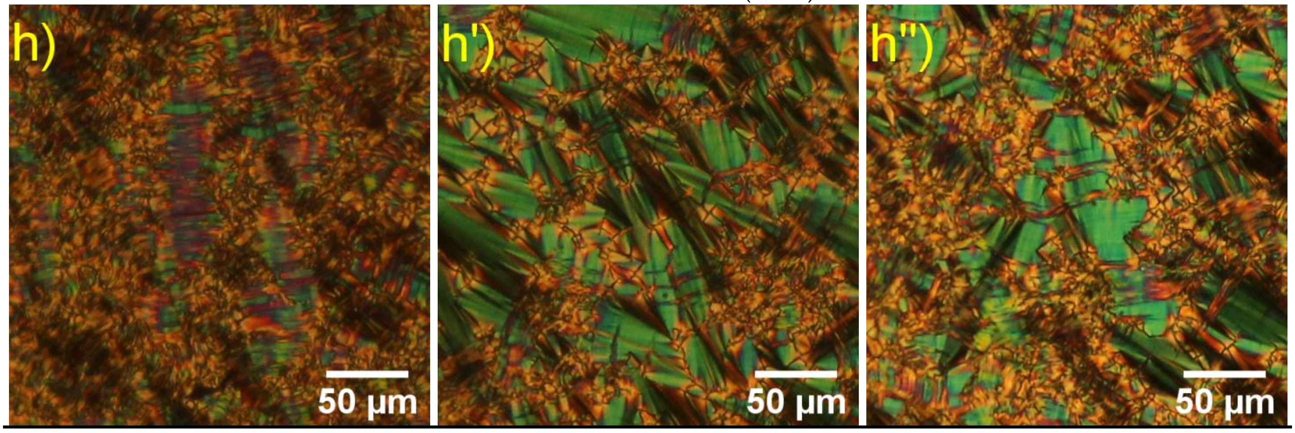

Figure S1. Textures registered during cooling with a rate of 2°C/min for Composites 1–3: (a-a'') SmA*, (b-b'') SmC*$_{\alpha}$, (c-c'') SmC*, (d-d'') SmC*$_{\gamma}$, (e-e'') SmC*$_A$ at 105°C, (f-f'') SmC*$_A$ at 90°C, and (g-g'') SmI* and (h-h'') coexistence two phases: SmI* and Cr$_1$. The orange perpendicular arrows in (a) represent the crossed polarizer and analyzer. Scale bars in the right lower corner are the same for each image (50 μm). All images were obtained from the same sample area in each Composite.

The considered focal conic defects are usually common when the molecules form a strong anchoring to the cell surface or other nucleation points (such nucleation points in the area between the glass plates can be, e.g., an admixtures or spacers, eliminating the thickness gradient in the cell used). Hence, the defects appear in random places on the recorded texture. Around the nucleation points, the molecules assume a radial orientation (the smectic layers thicknesses are preserved), and therefore, the smectic layers must curve locally (see Figure S2) around the nucleation centers [54]. Due to the liquid-like nature of the SmA* phase, adjacent nucleation centers grow and combine with each other to form Dupin's cyclides. When the two nucleation centers grow and meet, the outer molecules' layers form a junction [54]. A line of hyperbolic optical discontinuity is formed at the junction site. The molecules at a junction are disorganized, and between crossed polarizers, the considered area appears dark (dark cross). As Goodby reported, this defect type is easily formed when the cell thickness is more than 3 to 4 μm (in our case, about 5 μm) [54]. Figure S2 shows an example of the topological focal conic defect formation with a clear marking of the conic curves (ellipse and hyperbola) on the texture fragment for Composite 3, the SmA* phase, 140 °C showing the crossing of the above-mentioned curves, formulating the defect. The plane in which the hyperbola lies in the planar boundary conditions is parallel to the substrate cell planes [53].

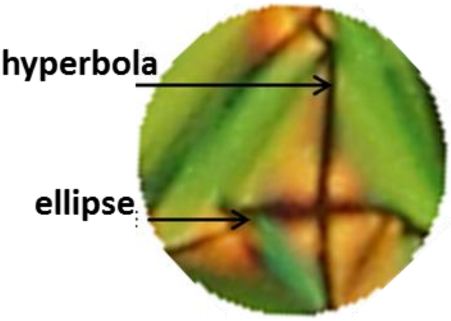

Figure S2. An example of the focal conic defect in the focal conic fan texture for the SmA* phase (Composite 3) together with conic curves (hyperbola and ellipse). In the measuring cell plane lies the hyperbola, the ellipse in the planar anchoring is perpendicular to the substrate planes (is a projection on the hyperbola plane). These areas are degenerated smectic layers.

In the inset (Figure S3a) is a table with ΔH values accompanied by the individual phase transitions. In addition to the increase in T_o for the SmI*–Cr_1 phase transition in Composite 3 versus Composite 1, the ΔH value increased significantly relative to pure (S)-MHPOBC. As can be seen from the table, the ΔH for this transition increased with the increase in the amount of Au nanoparticles. A similar behavior was observed for the SmC*$_A$–SmI* phase transition. On the other hand, for the Iso–SmA* phase transition, the situations for Composites 1–3 were completely different, and the value of ΔH decreased with the increasing concentration.

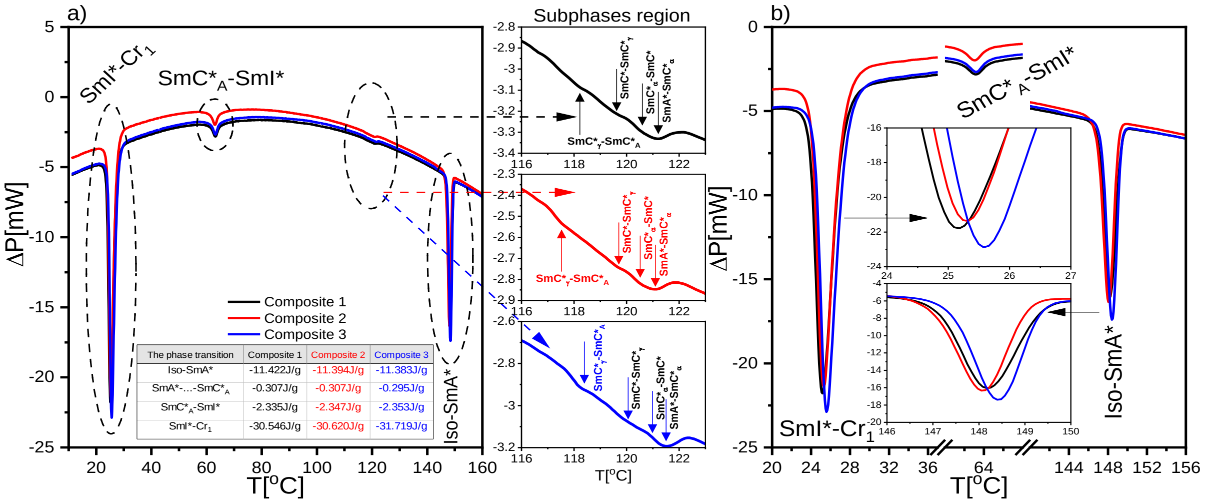

Figure S3. The calorimetric curves registered during cooling for Composites 1–3 with enthalpy changes for the chosen phase transitions (a). The insets in (a) present an enlarged temperature range of 116–126 °C. DSC curves were in the temperature range of the Iso–SmA* and SmI*–Cr_1 phase transitions (b).

Figure S4a shows the transmitted light intensity during the heating of Composites 1–3. Transitions Cr_1–Cr_2, Cr_4–SmC*$_A$, and SmA*–Iso are very clearly visible for all the studied samples. Based on these measurements, it can be concluded that Cr_1 is structurally very different from Cr_2–Cr_4, as evidenced by a clear decrease in the transmitted light intensity in the transition to Cr_2. The Cr_2–Cr_3 and Cr_3–Cr_4 transitions for Composites 2 and 3 are practically invisible optically, while, for Composite 1, only in the form of small humps (regime between 60 and 80 °C). This may indicate the influence of Au nanoparticles on the mentioned crystalline phases, e.g., by modifying the lattice parameters of these phases, which makes their structures very similar, and we did not see a difference between them in the optical measurements. Moreover, the Cr_1–Cr_2 transition temperatures were in agreement with those determined calorimetrically but only for Composites 1 and 2. We noted a significant discrepancy of this temperature for Composite 3 around 7 °C, which we do not fully understand. It was not excluded, as the heating rate was responsible for it. At high temperature change rates, nanoparticles can acquire a large amount of thermal energy in a very short time, becoming not only mobile but also capable of showing various movements at the molecular level, e.g., rotational (we should keep in mind that, apart from their sizes of 2–4 nm, they are additionally covered with an organic surfactant, and the surfactant kinetics under the influence of various temperature changes are unknown by us). Interestingly, within the SmC*$_A$ phase, we did not observe parabolic light intensity variations but only a monotonic decrease in the light intensity with the temperature, regardless of the admixture. Figure S4b shows the enlarged Cr_4–SmC*$_A$ phase transition range, while the vertical arrows do not correspond to the temperatures of this transition but the moment when only the SmC*$_A$ phase exists in the sample. For example, the temperature of the beginning Cr_4–SmC*$_A$ phase transition for Composite 3 from the optical method is about 83.0 °C (±0.5 °C), while, from the calorimetric method, is about

81.9 °C (0.1 °C). A temperature mismatch of 0.5 °C may result from different measurement conditions (aluminum and glass heat exchangers). In Figure S4c, the enlarged range of subphases, the arrows represent very subtle SmC*–SmC*$_\alpha$ phase transitions. In turn, in Figure S4d is the enlarged range between the crystalline phases with the largest structural differences. In the table (Figure S4e), we noted the SmC*–SmC*$_\alpha$ transition temperatures, which are the values at the maximum of the subtle jumps in Figure S4c.

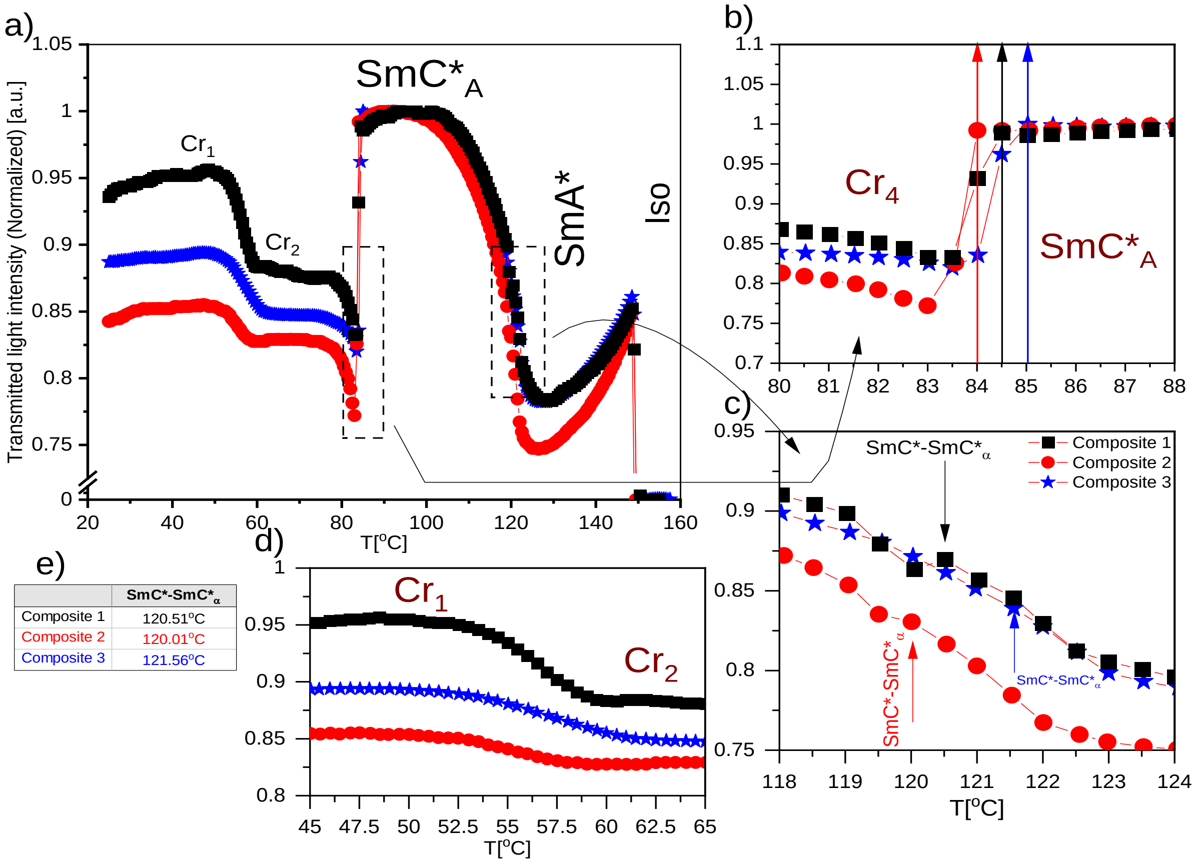

	SmC*-SmC*$_\alpha$
Composite 1	120.51°C
Composite 2	120.01°C
Composite 3	121.56°C

Figure S4. Temperature dependence of the normalized transmitted light intensity during heating (a) and the selected temperature range in the vicinity of the Cr_4–SmC*$_A$ (b), the SmC*–SmC*$_\alpha$ (c), and the Cr_1–Cr_2 phase transitions (d) and the SmC*–SmC*$_\alpha$ phase transition temperature for the Composites studied (e).

A characteristic feature of this hexagonal phase is the molecules grouping on the smectic layer in such a way that their gravity centers form hexagons with an additional molecule inside. The molecules in this phase are tilted to the hexagon apex at a certain angle, and if chiral, they form a helical superstructure. This superstructure is similar to the helical structure in the SmC*$_A$ phase; however, for the latter, there is no higher degree of center alignment in the smectic plane.

At the spectral range 430–455nm, the situation is a bit different. At 122 °C the fluorescence intensity is the lowest for Composite 1, while it increases with the increase of the admixture concentration. In turn, at 118 °C, a very intensive fluorescence is present in Composite 1. Interestingly, for Composites 1 and 2, the fluorescence is very poor at 88 °C, and it is decreases with the decrease in the temperature (at 68 °C, there is no fluorescence). Meanwhile, for Composite 3 at 68 and 88°C, the fluorescence is quite clear visible. This phenomenon is not well-understood by us. The mere Au nanoparticles present in the samples should give similar results for Composites 2 and 3; however, the microscopic observations are different for them. This situation did not exist for the 490–590-nm spectral range. It is not likely that Composites 2 and 3 at 68 °C were in different liquid crystal phases.

a)

Sample	68°C (SmC*$_A$)	88°C (SmC*$_A$)	118°C (SmC*$_\gamma$)	122°C (SmA*)
Composite 1	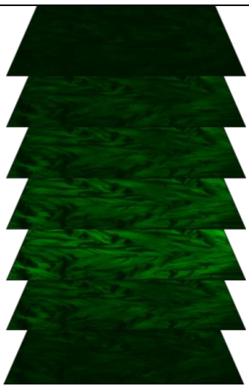	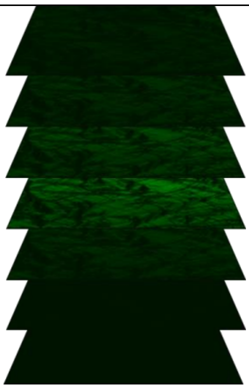	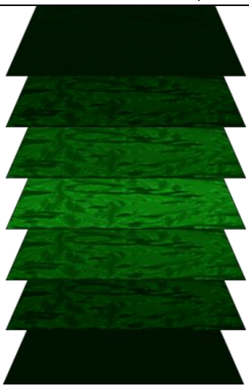	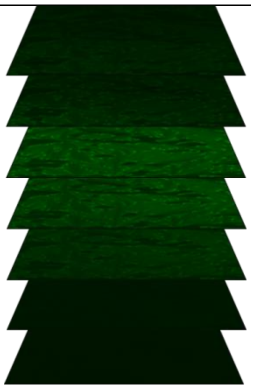
Composite 2	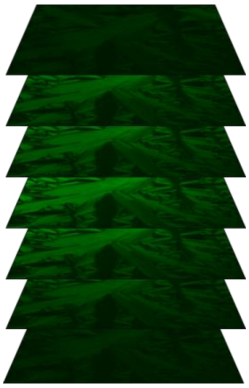	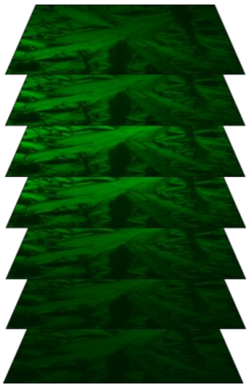	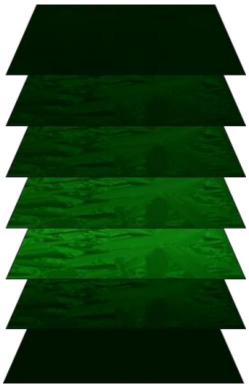	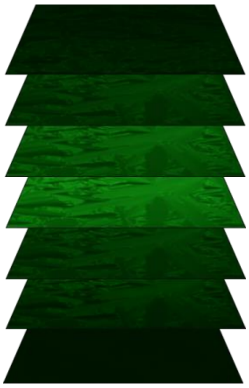
Composite 3	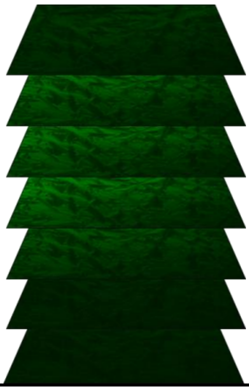	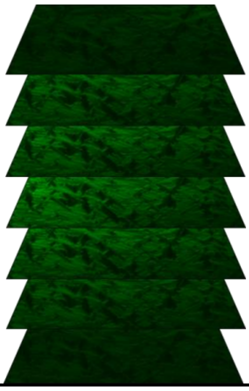	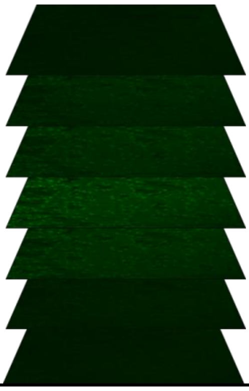	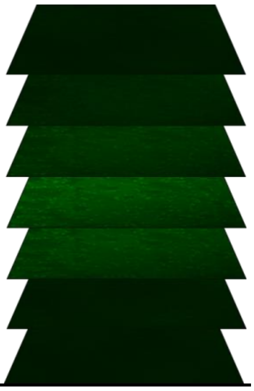

b)

Composite 1	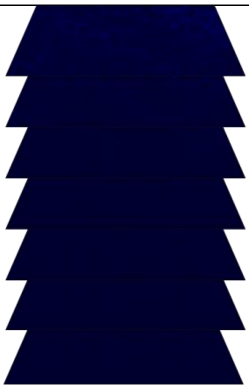	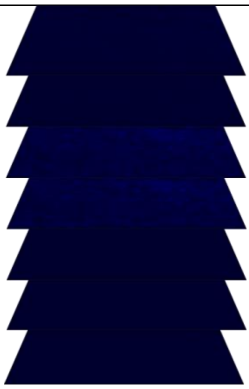	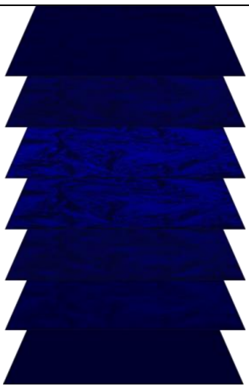	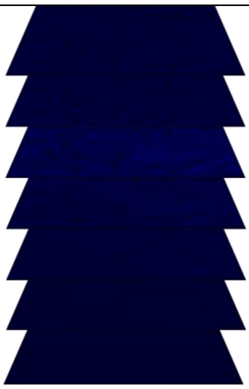

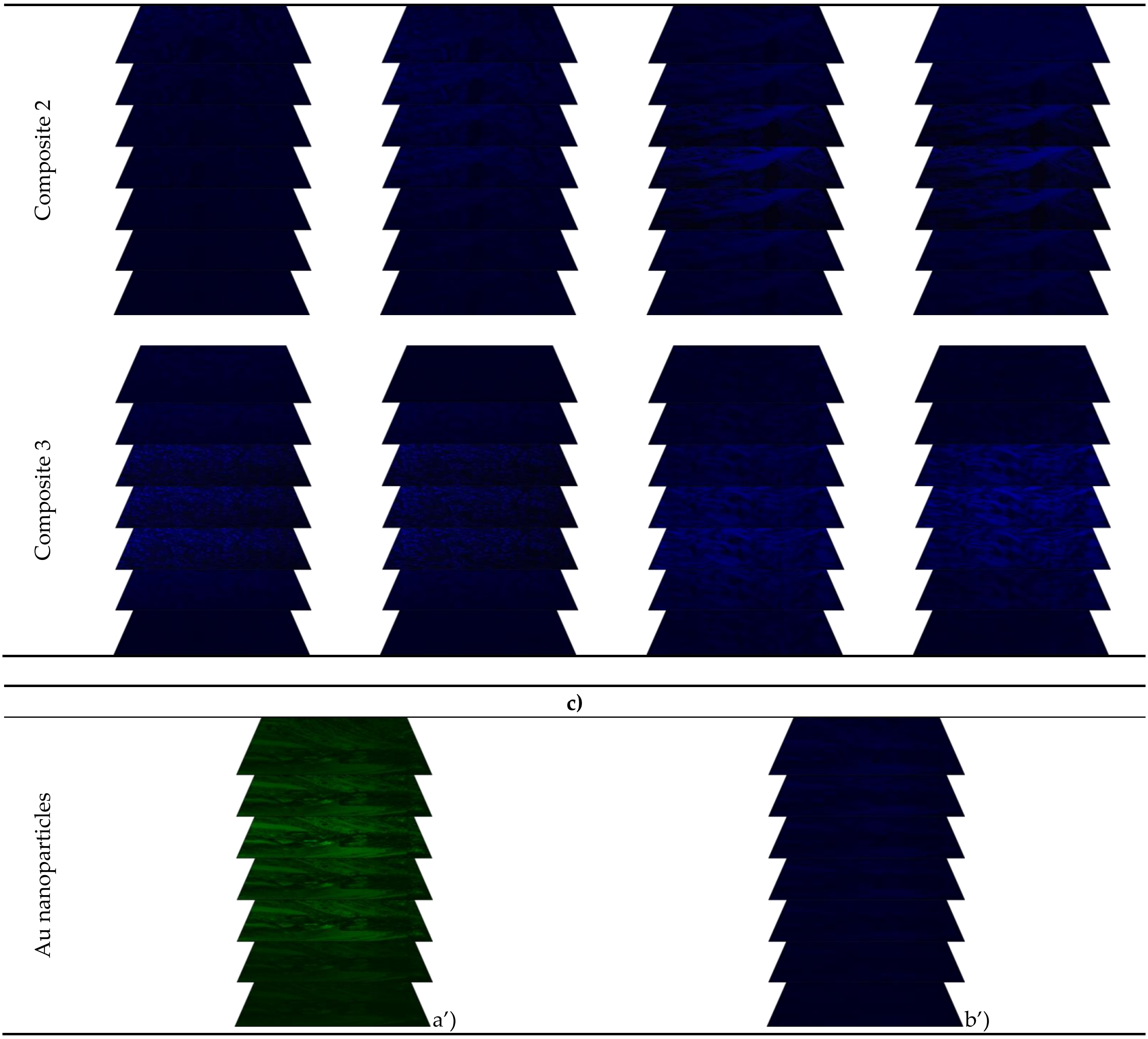

Figure S5. Fluorescence intensity in subsequent slices of the samples detected in the spectral ranges 490–590 nm (a) and 430–455 nm (b) at the chosen temperatures for Composites 1–3, as well as for the Au nanoparticles at 490–590 nm (a′) and 430–455 nm (b′) (c).

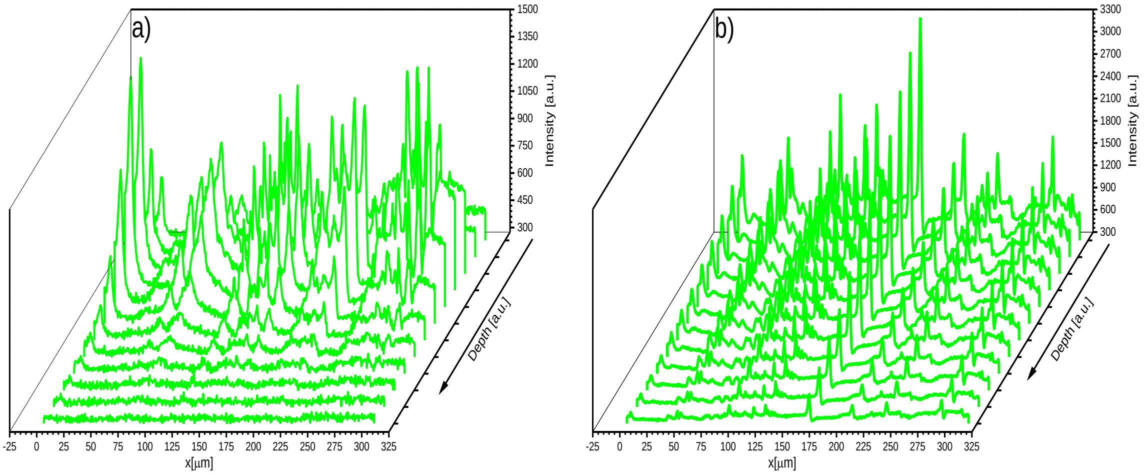

Figure S6. Fluorescence intensity of Composite 1 (a) and Composite 3 (b) detected in the green light range at 118 °C.

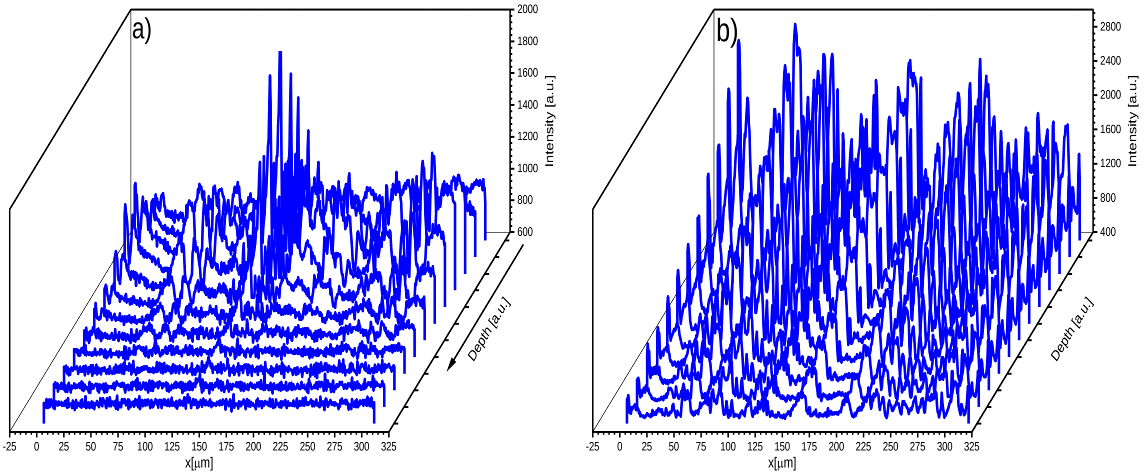

Figure S7. Fluorescence intensity of Composite 1 (a) and Composite 3 (b) detected in the blue light range at 118 °C.

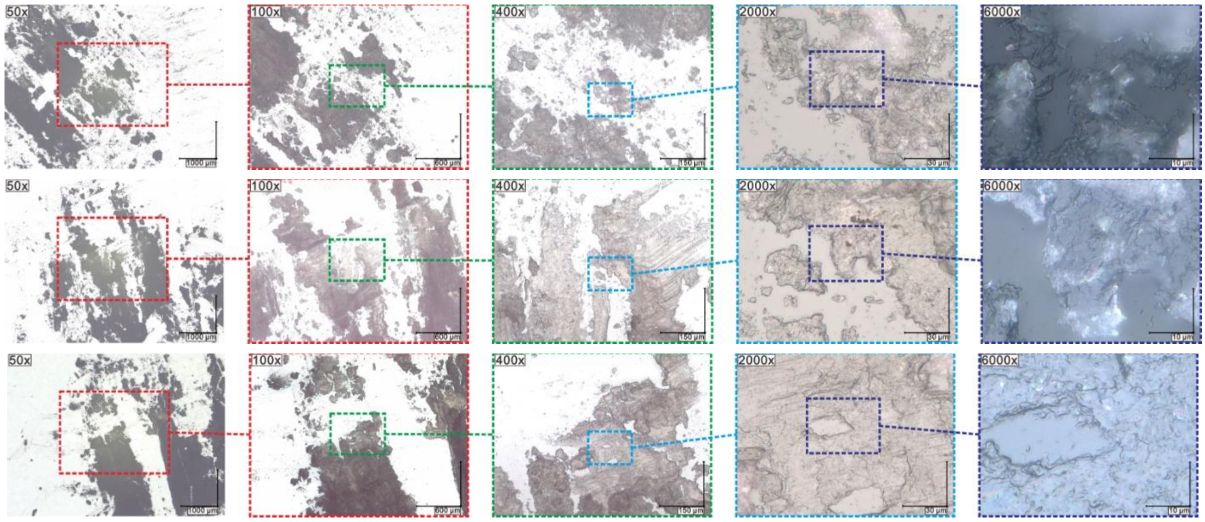

Figure S8. Room temperature digital optical images with coaxial illumination for pristine Composite 1 (the first row), Composite 2 (the second row), and for Composite 3 (the third row). The colored-dotted squares represent the enlarged areas.

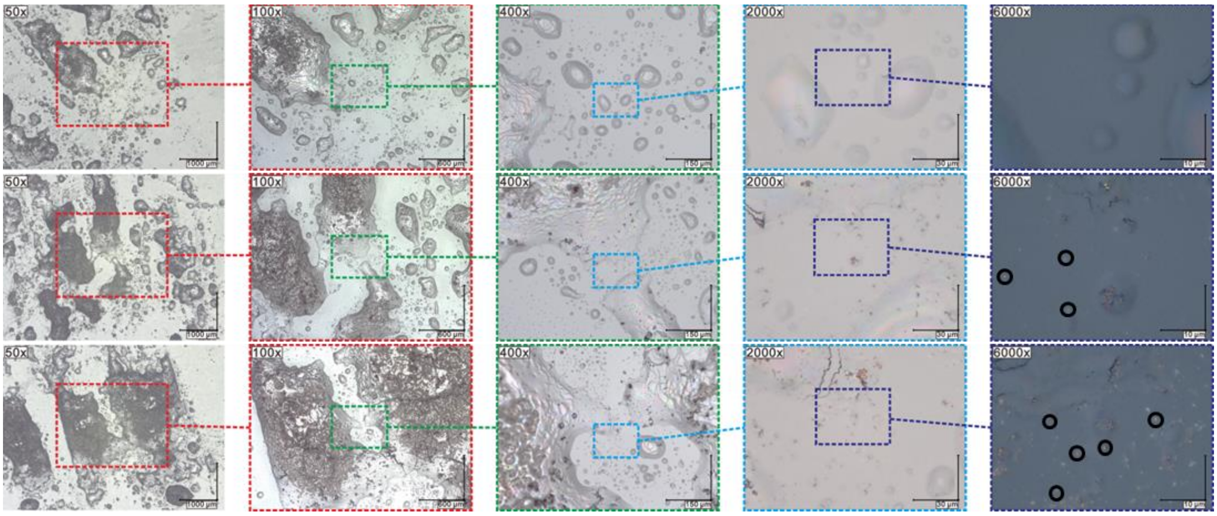

Figure S9. Room temperature digital optical images with coaxial illumination for Composite 1 (the first row), Composite 2 (the second row), and Composite 3 (the third row) cooled to room temperature after being heated to 155 °C and kept at this temperature for 5 minutes. The colored, dotted squares represent those enlarged. The black circles indicate white spots—small aggregates of Au nanoparticles.

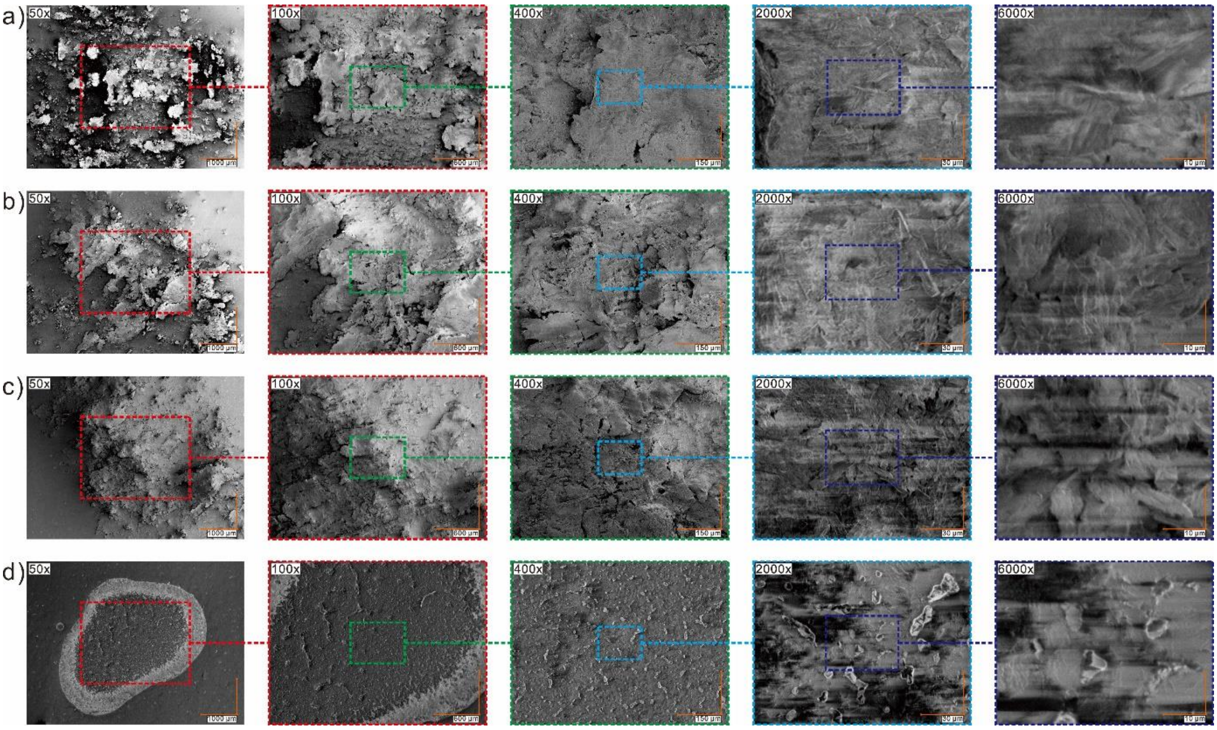

Figure S10. SEM images for Composite 1 (a), Composite 2 (b), Composite 3 (c), and the Au nanoparticles (d). The colored, dashed rectangles represent the enlarged areas.

Composite 1	Composite 2	Composite 3	Reference
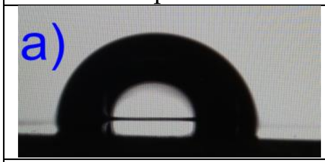	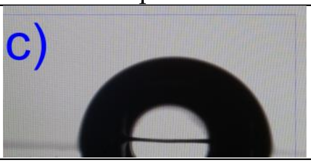	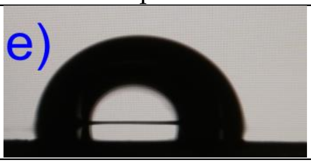	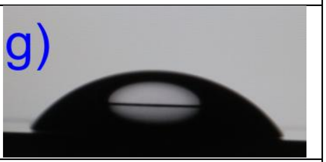
18°C Cr	18°C Cr	18°C Cr	18°C

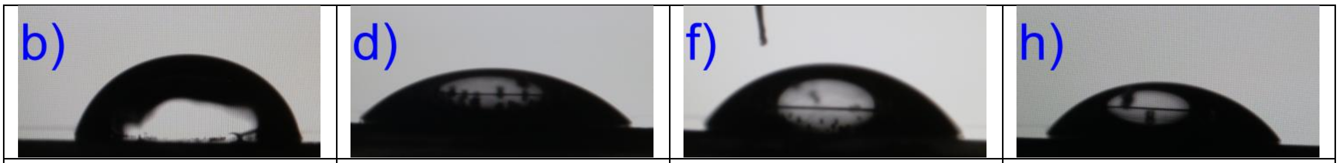

78°C Cr	87°C SmC^*_A	87°C SmC^*_A	87°C

Figure S11. Images for the water droplet deposited on the thin layer of Composites 1–3 and pure silicon wafer (reference) at the selected temperatures.

In the first stage, the switching times τ behavior to the amplitude of the alternating electric field was checked at two selected temperatures of the SmC^*_A phase, namely 117 and 105 °C. Measurements were made on completely disordered samples, and after each measurement series, the samples were heated to an isotropic liquid and subsequently cooled down to the next measuring temperature. Figure S10 presents the dependence τ(U) for all composites at 105 °C and f = 50Hz. As can be seen with the U value increase, a τ decrease, which was to be expected, because the higher electric field E causes the electric dipole moments to experience a higher force moment (the faster reorientation). The uncertainties related to the experimental points have different sizes, which results from the fact that, during the data collection, the time base on the oscilloscope was changing. Up to 120 V, it can be observed that Au nanoparticles do not influence the τ values. Above 120 V, it can be seen the Au nanoparticles lower the τ values, and for 150 V, they are equal to: 22, 30, and 31 μs for Composite 3, Composite 1, and Composite 2, respectively. The downward trend is much more noticeable for Composite 3 than for Composite 2. Only for Composite 3 above 120 V was there a continuous decrease in the τ value, while, for Composite 2 at 150 V, there was a subtle increase. In addition, as the alternating voltage amplitude increased, the sample response during the change in signal polarity became narrower in the time domain. The wide sample responses at a low U are because the molecules respond to a change in the signal polarity; however, their reorientation time distribution is large. This behavior was observed for all the samples. The inserts in Figure S10 show the dependencies of τ(U) at 117 °C for the two selected frequencies, namely 50 and 100 Hz. For both frequencies under consideration, at low U values up to about 110 V for 50 Hz and 100 V for 100 Hz, plateau regions are observed for τ. Only above these U values can we see a higher times variability. In the frequency of 50 Hz above 120 V, the shortest times are characterized by Composite 2 (about 25 μs) and the longest by Composite 1 (about 35 μs), while, for frequencies of 100 Hz and above 120 V, the shortest times are shown by Composite 3 (around 22.5 μs) and the longest for Composite 2 (around 35 μs). Moreover, in the 120–150-V range with both frequencies, only Composite 3 shows a plateau, while Composite 1 shows a typical downward trend. For Composite 2, sharp jumps in the τ values at 120 V and 140 V for 50 and 100 Hz, respectively, were recorded in this range. The decrease in the τ value for Composite 3 by about 7 μs and the increase for Composite 2 by about 7 μs with the increasing frequency are not fully understood by us. However, this behavior is very promising for the use of Composites 2 and 3 as electrical switches whose frequency dependencies depend on the dopant concentrations.

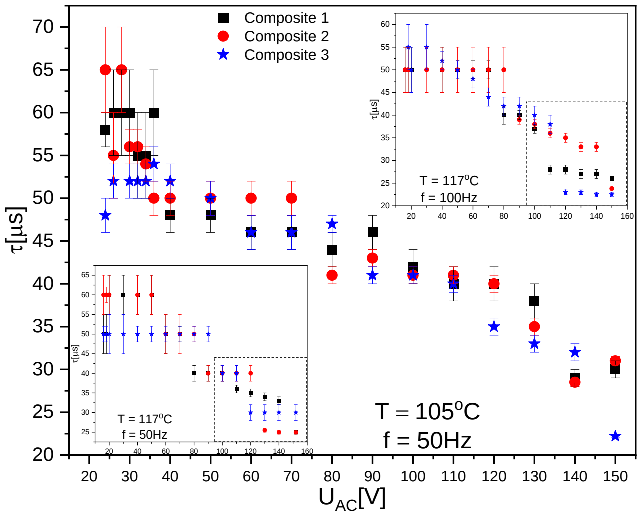

Figure S12. Amplitude dependence of the reorientation times τ for f = 50 Hz at 105 °C. The upper inset presents the results for f = 100 Hz at 117 °C. The bottom inset presents the results for f = 50 Hz at 117 °C. The dashed rectangles in the insets show the amplitude range where the changes are the highest.

In turn, although nanoparticles are decorated with aliphatic chains, which are the deliberate procedure to eliminate aggregation processes, we cannot completely exclude this parasitic process. Let the typical (covalent) atomic radius for gold be r ~ 135pm. The nanoparticles considered in this article are spherical clusters with a diameter of 2–4nm and, therefore, with radii of R ~ 1 to 2 nm. The single-atom volume is proportional to r, such as ~ r^3, while the volume of the whole nanoparticle is radius R = 1.5 nm, such as ~ R^3. Thus, the nanoparticles with radius R consist of $N \sim (R/r)^3 \approx 1372$ gold atoms. In turn, the number of atoms on the nanoparticle surface is $N - N' = N - ((R-r)/r)^3 \approx 338$. Therefore, theoretically, each nanoparticle with a radius of 1.5 nm has 338 Au atoms on the surface, and each such Au atom can chemically bond through a strong covalent bond with the sulfur atom, which is the surfactant part. Obviously, for steric reasons, not every Au atom can be bounded. It is possible that, with the increasing Au concentration, adjacent nanoparticles will contact metallic cores at certain points, or the metallic cores will be very close to each other. The more such cases there are, the more Θ will be modified.
